# New Development in Understanding Drug–Polymer Interactions in Pharmaceutical Amorphous Solid Dispersions from Solid-State Nuclear Magnetic Resonance

**DOI:** 10.1021/acs.molpharmaceut.2c00479

**Published:** 2022-08-29

**Authors:** Andrea Pugliese, Michael Tobyn, Lucy E. Hawarden, Anuji Abraham, Frédéric Blanc

**Affiliations:** † Department of Chemistry, University of Liverpool, Liverpool L69 7ZD, United Kingdom; ‡ Drug Product Development, Bristol-Myers Squibb, Moreton CH46 1QW, United Kingdom; § Drug Product Development, Bristol-Myers Squibb, New Brunswick, New Jersey 08903, United States; ∥ Stephenson Institute for Renewable Energy, University of Liverpool, Liverpool L69 7ZF, United Kingdom

**Keywords:** Amorphous solid dispersions (ASDs), solid state nuclear magnetic resonance (NMR), API−polymer interactions, ASD stability, hot-melt extrusions (HMEs), spray-drying (SD)

## Abstract

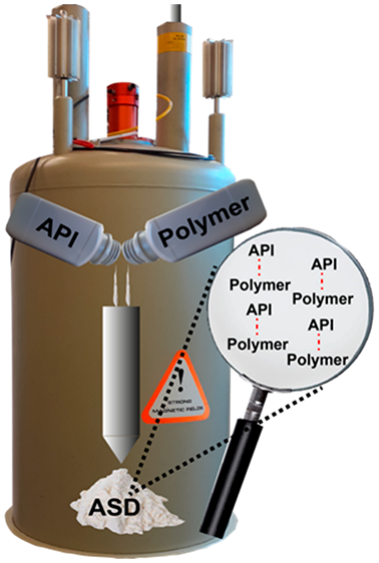

Pharmaceutical amorphous solid dispersions (ASDs) represent a widely used technology to increase the bioavailability of active pharmaceutical ingredients (APIs). ASDs are based on an amorphous API dispersed in a polymer, and their stability is driven by the presence of strong intermolecular interactions between these two species (e.g., hydrogen bond, electrostatic interactions, etc.). The understanding of these interactions at the atomic level is therefore crucial, and solid-state nuclear magnetic resonance (NMR) has demonstrated itself as a very powerful technique for probing API–polymer interactions. Other reviews have also reported exciting approaches to study the structures and dynamic properties of ASDs and largely focused on the study of API–polymer miscibility and on the identification of API–polymer interactions. Considering the increased use of NMR in the field, the aim of this Review is to specifically highlight recent experimental strategies used to identify API–polymer interactions and report promising recent examples using one-dimensional (1D) and two-dimensional (2D) experiments by exploiting the following emerging approaches of very-high magnetic field and ultrafast magic angle spinning (MAS). A range of different ASDs spanning APIs and polymers with varied structural motifs is targeted to illustrate new ways to understand the mechanism of stability of ASDs to enable the design of new dispersions.

## 1 Amorphous Solid Dispersions

The formulation of low solubility crystalline active pharmaceutical ingredients (APIs) or drugs in the amorphous form is a recognized robust methodology exploited to improve their dissolution rates and bioavailability.^1,2^ Amorphous solid dispersions (ASDs) can be used for this purpose and others, such as the enhancement of stability when salt forms of an API are unstable. Several approaches have been developed to stabilize ASDs among which hot-melt extrusion (HME) and spray-drying (SD) technologies are the most widely used and the only ones used commercially by the pharmaceutical industry.^3−5^ ASDs are formulations of one (or more) active ingredient in the amorphous state stabilized by inert and hydrophilic carrier(s) or matrix in the solid state (usually a polymer and/or additive) aimed at obtaining a fully miscible, amorphous, and physically stable dispersion.^6,7^

**Figure 1:**
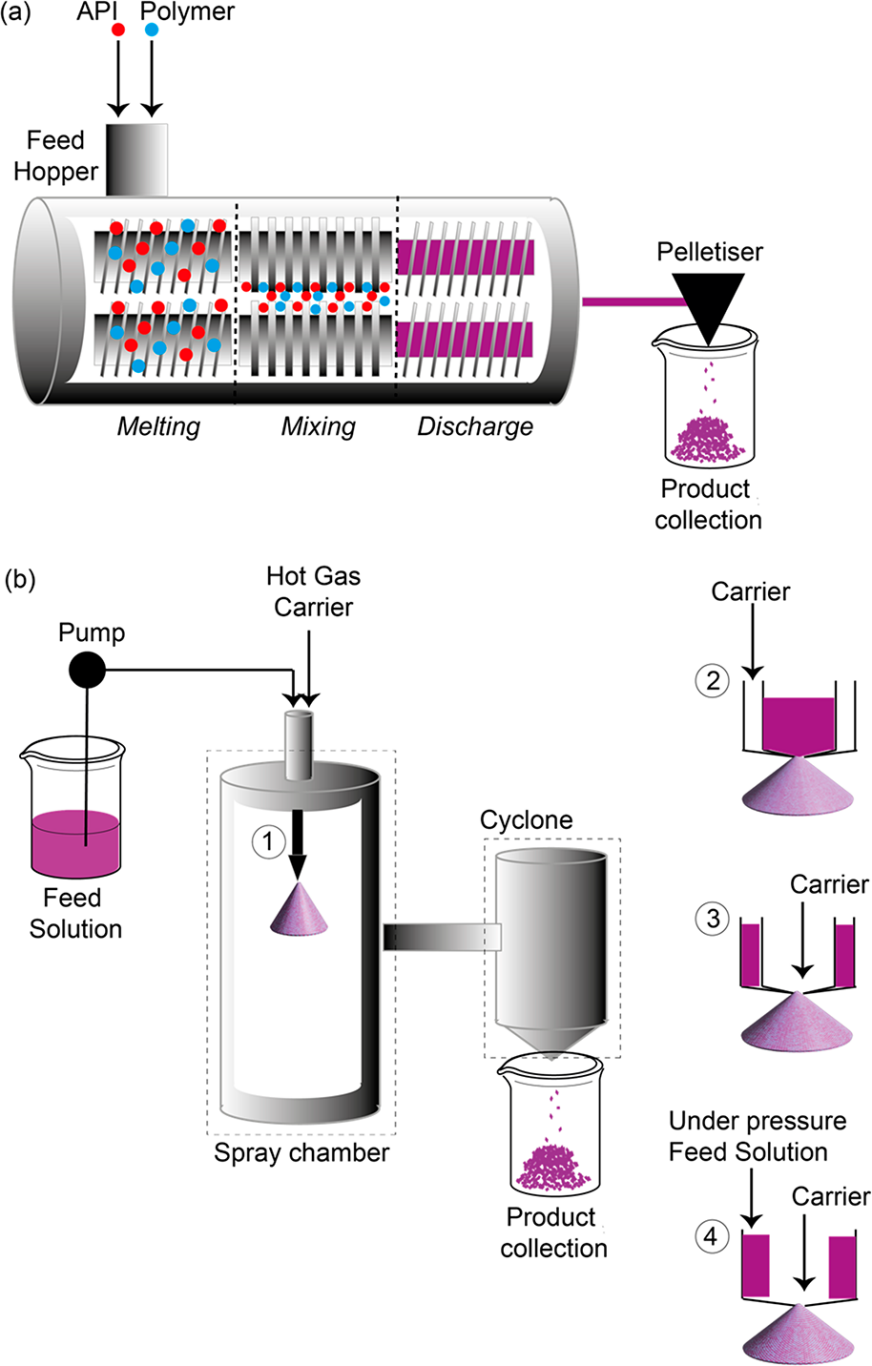
Schematics of (a) HME and (b) SD technologies for the preparation of ASDs. In (a), the three main steps are melting, mixing, and discharge where the material cools down and leaves the apparatus. Physical–chemical properties of the final product can be tunable using different screw designs and speeds and by controlling the temperature. In (b), the spray nozzle (component 1) of the chamber is available in several designs (components 2–4) based on the required specific applications. Figure adapted with permission from ref (5). Copyright Elsevier (2021).

HME is utilized extensively for commercial-scale ASD manufacturing because of several factors: it is a mature and a well understood process and can therefore be scalable and have a low cost; it is solvent free and thus environmentally friendly, and it operates as a continuous process well suited for large scale production.^2,8^ This technology contains several steps as illustrated in Figure 1a in which the drug and the polymer are mixed, melted, dispersed, and extruded under specific conditions, offering the flexibility to be tailored to a range of APIs, matrixes, and other excipients while also being suitable to oxygen sensitive and hydrolyzable drugs.^3^ However, two of the most important drawbacks of this technology are the high energy consumption of the process and the heating processes, which preclude the formulation of thermolabile APIs.^5^

Another widely used technology to commercially manufacture ASDs in the pharmaceutical industry is the spray-drying (SD) methodology that also offers a scalable process; however, scaling up is more complex with SD than with HME.^4^ The SD process (Figure 1b) requires an initial API–polymer solution/suspension in a system that might contain water as the feed solution, which is then spray dried through a spray-nozzle (component 1 in Figure 1b) for which various design exists (components 2–4 in Figure 1b) to accommodate more than one feed or high pressure.^5^ Upon contact of the feed solution droplets with the hot gas/air carrier, the solvent system evaporates quickly, leading to dried ASD particles that are separated by the gas stream in the cyclone and then collected. Once gathered, the particles are subjected to a secondary drying process to remove solvents and reduce any residual moisture to an appropriate level. The spray-dried systems can then be formulated into a conventional tablet system.

Polymers in ASDs play a key role in stabilizing the thermodynamically metastable nature of the amorphous API. Polymers raise the inherent glass transition temperature (*T*_g_) of the system, which effectively reduces the amorphous molecule’s mobility, making it less likely that it will encounter other molecules to trigger the crystallization process. Further amorphous stability enhancements can be achieved by specific chemical interactions between the drug and API species to further reduce the molecular mobility of the amorphous drug and increase the *T*_g_ of the formulation.^9^ The formation of the drug–polymer intermolecular interactions, such as hydrogen bonding (H-bond), ionic forces, π–π, or electrostatic interactions, are well established as the most significant interactions capable of stabilizing dispersed systems^8^ by inhibiting recrystallization phenomena in the amorphous matrix and preventing competitive API–API or polymer–polymer intramolecular interactions. The identification and the understanding of the physical stability of ASDs remain a significant challenge and open exciting future perspectives for the design of ASDs stabilized by suitable and tunable API–polymer interactions.^10^

Historically, thermal analysis methods, such as differential scanning calorimetry (DSC) and temperature-modulated DSC, have often been employed to elucidate API–polymer interactions in ASDs, which allows *T*_g_ measurements from the miscibility of the various ASD components to be inferred.^11,12^ One such approach is the Gordon–Taylor model^13^ that estimates the *T*_g_ of an ideal binary mixture (*T*_g,mix_), where significant deviations between the predicted *T*_g,mix_ and experimentally determined *T*_g_ provide useful information about the interactions between the various constituents of the mixture and potentially repulsive interactions, which destabilize the system.^14,15^

A range of analytical methods including vibrational, such as Raman, and Fourier-transform infrared (FT-IR) spectroscopies have been used to provide atomic scale information about solid dispersions. Raman applications specifically include the measurements of crystallization rate,^16^ while confocal Raman microscopy and Raman imaging have been employed in mapping solid dispersions to identify and discriminate crystalline/amorphous domains,^17^ thereby providing indirect information about the existence of API–polymer interactions. Evidence of recrystallization phenomena can be observed from changes in band wavelength and a comparison of band intensity ratios or studies of spatially time-resolved Raman generated using multivariate curve resolution that leads to monitoring the evolution of an amorphous drug in an ASD. FT-IR methods probe the H-bond in specific functional groups such as hydroxyl, amino, and carbonyl groups and permit identification of this specific interaction in the API and/or the polymer.^18^ It has been shown that, when those functional groups are involved in H-bond interactions, a simultaneous decrease in the stretching frequency and a widening of their absorption bands are observed due to smaller intermolecular distances between the donor–acceptor groups.^19^

The wide use of these techniques is justified by their versatility (obtaining information on the internal energy of the samples, API–polymer miscibility, and the presence of molecular interactions), the small amount of sample required, their ease of use, and their relatively short analysis time. However, some drawbacks exist and include the presence of moisture for FT-IR data, the photodegradation of the sample in Raman spectroscopy, or the experimental conditions (DSC and modulated DSC), which require careful consideration.^200^

Solid-state nuclear magnetic resonance (NMR) has emerged as a significantly powerful tool to access structural and dynamics information across the biological, chemical, material, and physical sciences.^20,21^ In particular, despite the high cost of the equipment, NMR is arguably the most powerful approach to obtain structural information at the atomic level and is complementary to the analytical techniques mentioned above. NMR plays an important role in pharmaceutical sciences^22,23^ to enable the structural understanding of the API^24^ and polymer,^25,26^ identify crystalline polymorphs,^27^ monitor drug recrystallization phenomena from amorphous systems, and understand API–polymer interactions in ASDs.^28−38^ Although a number of reviews has been published on these topics, these papers report either broad overviews^39−41^ or specific applications in the field (e.g., ^19^F).^42^ This Review aims to describe the use of solid-state NMR to the understanding of the stability of ASDs based on the presence of API–polymer interactions and highlights the most modern NMR methodologies to achieve this. The purpose of this Review is to discuss the recent exciting literature in the field; the interested readers are referred to several excellent monographs for the basics of NMR^43−46^ and in the solid state.^20,47,48^ This Review specifically reports experimental NMR approaches to identify the presence of inter- and intramolecular API–polymer interactions in ASDs prepared by HME or SD technologies with examples based on a range of structurally different drugs and polymers.

We first show that one-dimensional (1D) experiments can be employed to highlight the presence of API–polymer interactions based on changes in chemical shifts for certain signal(s) in the spectra of the ASD compared with those of the amorphous API or polymer. 1D experiments are also useful to identity API recrystallization processes from line shape analysis and relaxation measurements. We then reveal that the nature of the API–polymer interactions can be obtained from two-dimensional (2D) homo- and heteronuclear NMR correlations. In particular, we exemplify the ^1^H–^13^C/^19^F heteronuclear correlation (HETCOR) and ^1^H–^1^H dipolar double quantum (DQ) correlation as methods to identify site-specific intermolecular contacts between the API and polymer. Finally, we illustrate how 2D experiments carried out at ultrafast magic angle spinning (MAS) frequency (>60 kHz) and very-high magnetic field (>600 MHz, 14.1 T) and involving quadrupolar and/or low gyromagnetic nuclei (i.e., ^14^N) can be used to probe API–polymer interactions.

## 2 Chemical Shift as a Unique Observable of the API–Polymer Interaction

The most straightforward experimental approaches currently used to characterize novel ASDs often include 1D NMR spectra data collection involving most, if not all, NMR active isotopes. This is typically accomplished by recording ^1^H, ^19^F, ^13^C, and ^15^N NMR nuclei (as applicable) in which the chemical shielding interactions have been demonstrated to be very sensitive to subtle changes in the local electronic environment, indicating the presence of API–polymer interactions in ASDs.

Solid-state NMR spectroscopy involves powder samples consisting of many randomly oriented crystallites; hence, the nuclear spin interactions, such as chemical shielding, dipole–dipole coupling, and in cases involving a nuclear spin larger than 1/2 (e.g., ^14^N), quadrupolar coupling, are all orientation dependent (or anisotropic). This results in a range of resonance frequencies leading to NMR signal broadening.^20,47^ These anisotropic interactions (except the quadrupolar one) can be successfully averaged out by magic angle spinning (MAS), in which the sample is spun at an angle of 54.7° (magic angle) with respect the direction of the static magnetic field. Under conditions where the MAS frequency is on the same order of magnitude (or greater) than the NMR interactions, the broadened resonances observed in static (non spinning) solid-state NMR spectra largely vanish to yield narrower lines.

The direct observation of ^1^H and ^19^F NMR signals has been used to detect polymorphs in crystalline samples and to distinguish multiple APIs in ASDs and has allowed the identification of molecular interactions between various components of the dispersion from chemical shift changes.^8,39^ In addition, in 1D experiments, the comparison of line widths can be used to discriminate crystalline API from amorphous API as the latter experiences severe inhomogeneous line broadening arising from a large distribution of randomly distributed chemical environments,^39,49^ an effect that is not averaged out by MAS. The low receptivity of ^13^C and ^15^N arising from both low natural abundance (1.1% and 0.4%, respectively) and poor intrinsic sensitivity can significantly be overcome by the transfer of polarization from highly receptive nuclei (usually ^1^H or ^19^F). In the solid state, this now routine approach is called cross-polarization (CP) in which the polarization transfer is radio frequency driven by heteronuclear dipolar coupling.^48^ This allows for spectra with higher signal-to-noise ratios to be obtained in a reasonable amount of experimental time (hours) for most ASDs even at relatively high API loading.

**Figure 2:**
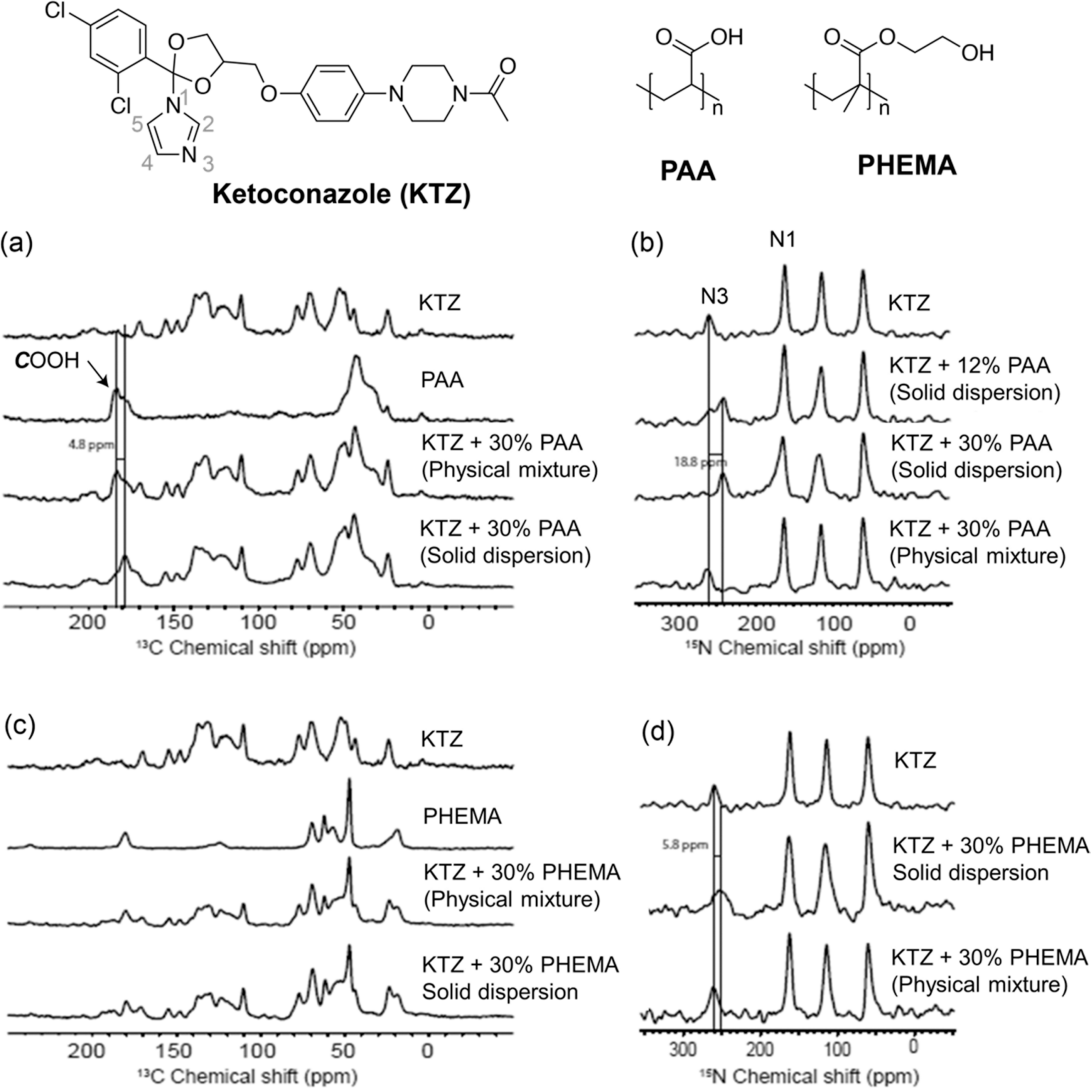
Comparison of the (a, c) ^13^C and (b, d) ^15^N CP MAS NMR spectra of amorphous KTZ, PAA, and PHEMA polymers, their physical mixtures, and ASDs. The vertical black lines highlight differences in chemical shifts in the ASDs with respect to the individual components, indicating the presence of API–polymer interactions. The chemical structures of KTZ and polymers are shown at the top of the figure. Reprinted from ref (28). Copyright 2015 American Chemical Society.

Figure 2 shows 1D ^13^C and ^15^N CP spectra that were successfully exploited to highlight H-bond interactions in ASDs between ketoconazole (KTZ) and a range of different polymers (such as poly(acrylic acid) (PPA), and poly(2-hydroxyethyl methacrylate) (PHEMA)) with API concentrations ranging between 4 and 12 wt %.^28^ The ^13^C CP MAS spectrum (Figure 2a) of the KTZ-PAA ASD reveals that the ***C***OOH signal of PAA experiences a change of shift to lower frequency relative to the physical mixture and PAA itself, which can be attributed to the disruption of the dimeric H-bond of PAA, hence indicating that ***C***OOH is involved in stabilizing the amorphous API. The ^15^N CP MAS spectra of KTZ-PAA ASDs at different PAA loadings (Figure 2b) show the existence of two N3 imidazole nitrogen signals at around 260 and 240 ppm that can be attributed to “free” KTZ and to a N3 site engaged in a H-bond, respectively, as this 20 ppm change in shift is typical of H-bond interactions and supports that the most basic imidazole N3 nitrogen is engaged in stabilizing KTZ.^50^ No changes in ^15^N chemical shift are observed for the other imidazole nitrogen N1 signal or the piperazine nitrogens, highlighting that the KTZ-PAA H-bond interactions occur exclusively between the most basic imidazole N3 nitrogen and the ***C***OOH PAA.

While no changes in ^13^C chemical shift are observed between KTZ, KTZ-PHEMA ASD, and the physical mixture (Figure 2c), the ^15^N chemical shift of the N3 nitrogen KTZ in KTZ-PHEMA ASD is observed at a lower frequency than in KTZ itself and indicates that this site is involved in the formation of the API–polymer interaction likely with the −OH group of PHEMA.

**Figure 3:**
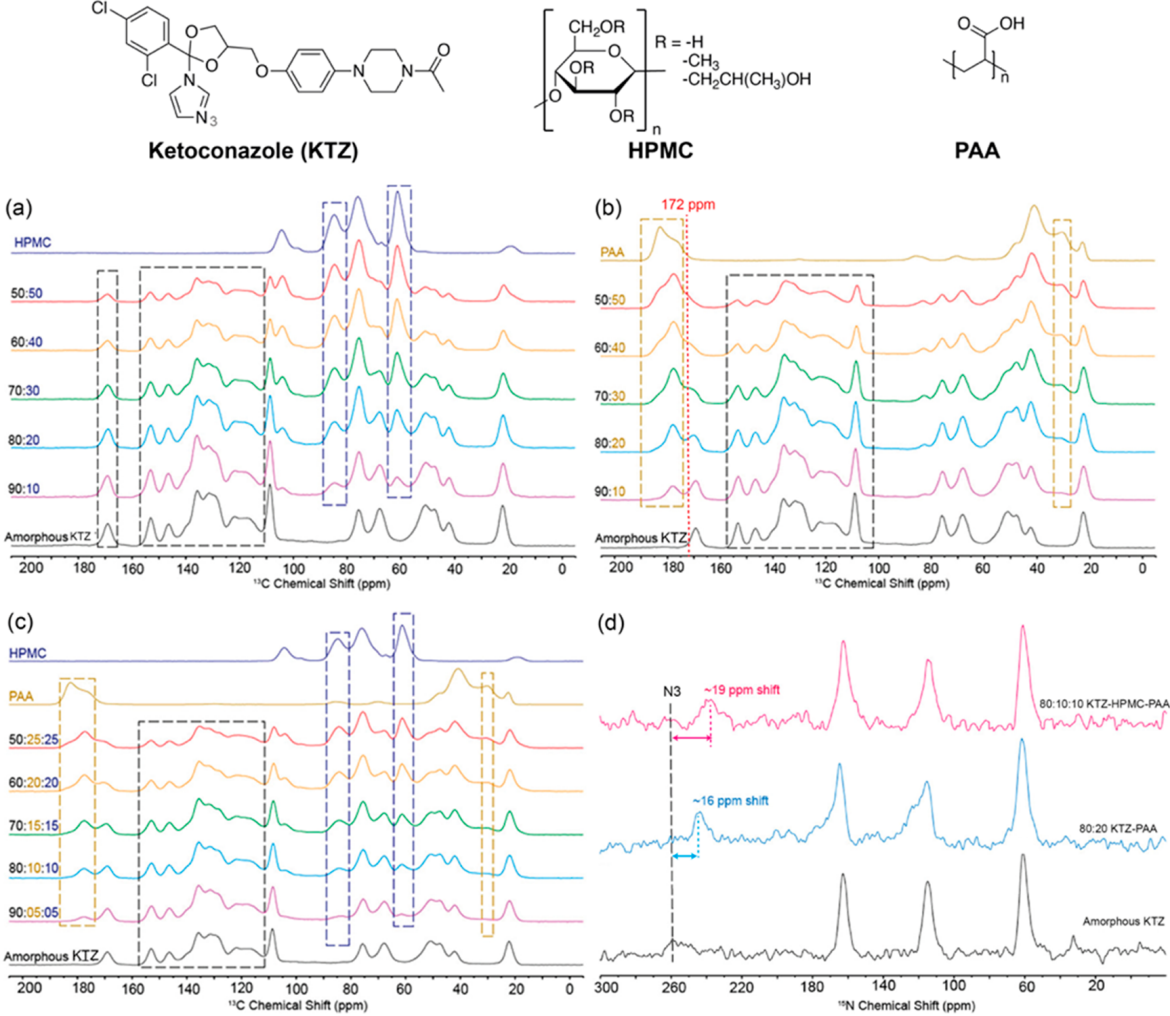
^13^C CP MAS spectra of (a) KTZ-HPMC, (b) KTZ-PAA, and (c) KTZ-HPMC-PAA ASDs at different drug loadings. (d) ^15^N CP MAS spectra of amorphous KZT, KTZ-PAA, and the ternary KTZ-HPMC-PAA ASDs. Chemical structures of KTZ, HPMC, and PAA are given on the top of the figure. Reprinted from ref (51). Copyright 2020 American Chemical Society.

Recently, a new class of ASDs has emerged, where a second polymer is added to the formulation. The capability of NMR to characterize this complex ternary ASD has been demonstrated using KTZ-HPMC-PAA ASD.^51^ While no changes in chemical shift were highlighted in the ^13^C spectra for the binary system KTZ-HPMC ASDs (Figure 3a) and the possibility of an interaction between the compounds was excluded, ^13^C spectroscopic data for both KTZ-PAA and KTZ-HPMC-PAA dispersions (Figure 3b,c) showed some interesting similar spectral features. In both systems, the intensity of the peak corresponding to the PAA dimer form (at 182 ppm) decreases with respect to the free form (at 177 ppm); the intensity across the drug loading changes and, interestingly, a new peak at 172 ppm emerges, which corresponds to hydrogen bonded KTZ. The changes in chemical shift have also been highlighted in the ^15^N spectra for both the binary KTZ-PAA and the ternary KTZ-HPMC-PAA ASDs (Figure 3d), suggesting a proton transfer or salt formation.^32^ These results probe the presence of ionic and H-bond interactions in both binary KTZ-PAA and ternary KTZ-PAA-HPMC ASDs, supported, respectively, by the loss of PAA dimers resulting from the KTZ-PAA interaction and by the presence of the signal at 172 ppm, which indicates the presence of hydrogen bonded KTZ.

Acid–base interactions are also known to significantly contribute to API stabilization in ASDs and are often much stronger than H-bonds, therefore offering exciting opportunities and attracting significant interests. However, only a few acidic polymers in the pharmaceutical armory are known, and they include PAA, hydroxypropylmethylcellulose (HPMC), hydroxypropylmethylcellulose acetate succinate (HPMC-AS), and poly(methacrylic acid-*co*-ethyl acrylate). These are, however, weakly acidic and not prone to protonate weakly basic moieties in APIs.

**Figure 4:**
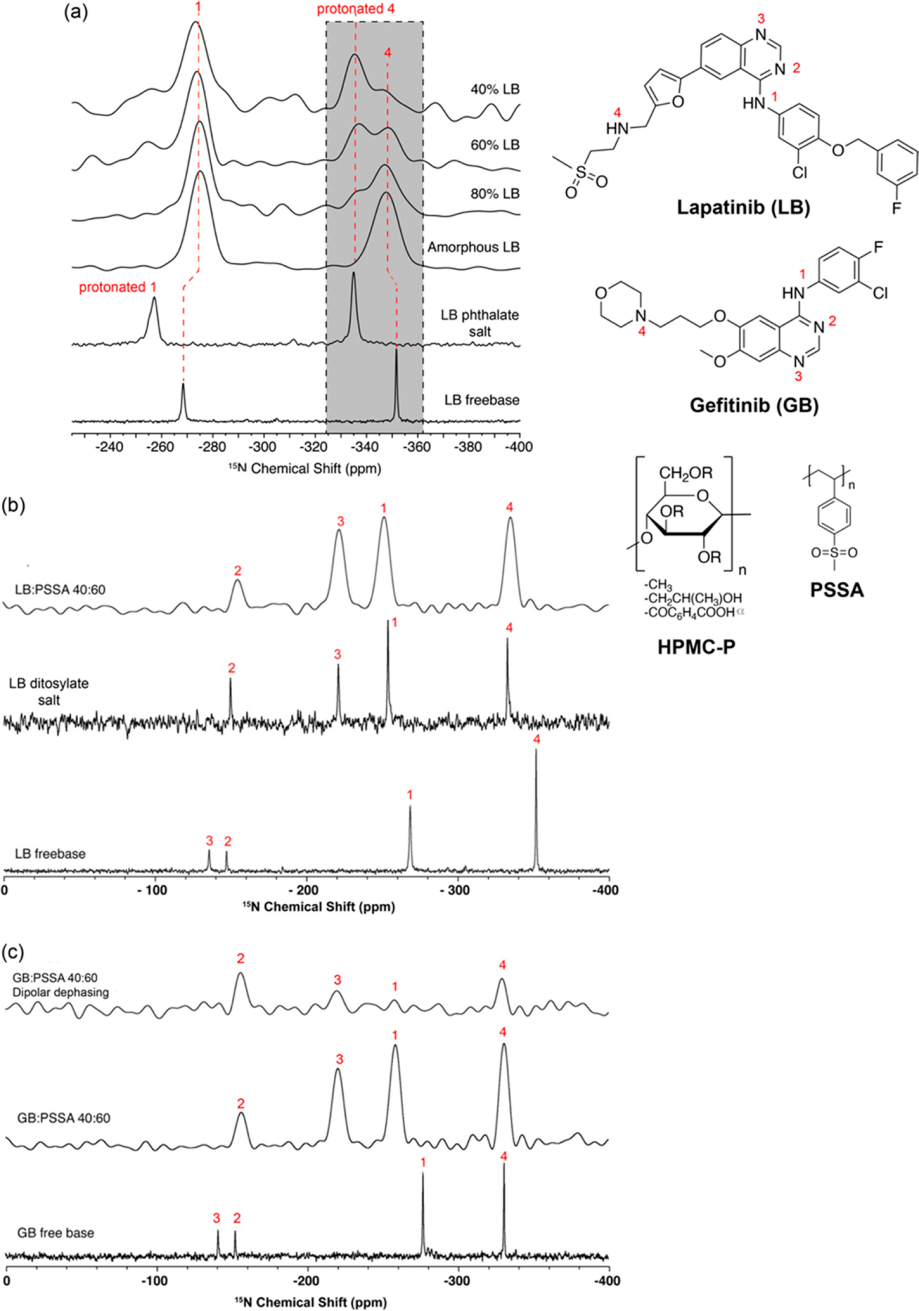
(a) ^15^N CP spectra of 40, 60, and 80 wt % LB-HPMC-P ASDs, amorphous LB, LB phthalate salt, and free base drug. The peak at −335 ppm in the ASD’s spectrum indicates the presence of a protonated amino group, hence highlighting the presence of an API–polymer ionic interaction. Reprinted from ref (29). Copyright 2015 American Chemical Society. (b) ^15^N CP spectra of LB free base, LB ditosylate salt, and 40 wt % LB-PSSA ASD. The large change in chemical shifts observed for N2 and N3 between the LB free base and the ASDs indicates protonation. (c) ^15^N CP spectra of gefitinib (GB) free base, 40 wt % LB-PSSA ASD, and the ^15^N dipolar dephased spectrum of LB-PSSA ASD. Spectral assignments and chemical structures of LB, GB, HPMC-P, and PSSA are given in the figure. Reprinted from ref (30). Copyright 2016 American Chemical Society.

Polystyrene sulfonic acid (PSSA) polymer, which is currently not a pharmaceutically approved polymer, is a promising inhibitor of recrystallization for several APIs in ASDs by forming strong acid–base interactions.^52^ Given that both lapatinib (LB) and gefitinib (GB) contain a range of basic nitrogen containing moieties such as anilinoquinazoline and amino groups, ^15^N NMR spectroscopy enables the identification of their acid–base interactions with HPMC-phthalate (HPMC-P) and PSSA acidic polymers (Figure 4)^29,30^ by taking advantage of the sensitivity of ^15^N chemical shifts to strong H-bond and protonation.^53^ The comparisons of the ^15^N CP MAS NMR spectra of crystalline LB free base and crystalline LB phthalate salt with their amorphous LB and LB-HPMC-P ASDs (Figure 4a) clearly show two peaks at around −350 and −335 ppm in the ASD that are attributed to the free amino N4 and protonated N4 signals, respectively. Therefore, this indicates the presence of an acid–base interaction between the protonated N4 in LB and HPMC-P, presumably via the −COO^–^ group, and reveals a significant change of the chemical shift of around 17 ppm for the amino (N4) signal (see gray shaded area), as expected between the LB phthalate salt (in which the N4 site is protonated) and free base. While the N4 amine peak in the ^15^N spectrum of amorphous LB appears at around −348 ppm as a single broad peak, the corresponding spectrum of the LB-HPMC-P ASD shows the presence of two clear peaks at around −350 and −355 ppm, attributable to the free amorphous amino N4 signal and protonated N4, respectively, as per comparison with the spectra of amorphous LB and LB phthalate salt reference samples. The existence of the protonated amine in the ASDs almost certainly means that a solid solution of the protonated LB at the N4 unit in the negatively charged polymer exists. Interestingly, for the HPMC-P dispersion, the position of the N1 aniline peak does not experience any significative changes. This suggests that the N1 sites are not involved in any interaction and confirms that this nitrogen is not protonated in the dispersions.

A similar approach was used to probe acid–base interactions in LB-PSSA and GB-PSSA ASDs, and the corresponding ^15^N CP MAS NMR^29,30^ spectra of LB, GB, and ASDs with PSSA (Figure 4b,c) reveal important contributions to the API–polymer interactions.^53^ In both LB- and GB-PSSA ASD, the ^15^N NMR signals of the quinazoline N sites (N3 both in LB and in GB) undergo a large shift of ∼80 ppm to a higher frequency with respect to crystalline LB/GB, indicating preferential protonation of this more basic quinazoline N site. Moreover, protonation of both amine N1 and N4 signals in LB-PSSA ASD is evidenced by their similar ^15^N shifts vs LB salt, confirming double protonation of the API by PSSA and strong acid–base interactions.

The presence of the API–polymer interactions in the GB-PSSA ASD established above involving the quinazoline N site is not linked to a change in ^15^N chemical shift of the ternary amine N4 (Figure 4c). Nevertheless, evidence of N4 being involved in the API–polymer interaction is supported by dipolar dephasing (interrupted decoupling) experiments^54^ that selectively select nuclear spins strongly coupled to ^1^H. The corresponding ^15^N spectrum of the 40 wt % GB-PSSA ASD highlights a lower signal intensity of the N1, N3, and N4 signals vs N2, confirming their protonation.

Acid–base interactions have also been identified in the indomethacin (IMC)–methacrylate copolymer Eudragit E (EE) ASDs solely from ^15^N NMR spectra^32^ while overlapping ^13^C signals between API and polymer prevented changes in chemical shifts to be observed. The ^15^N CP MAS NMR spectra of the 20–60 wt % IMC-EE ASDs exhibit two peaks at around −360 ppm, attributable to the EE amino signal, and at around −344 ppm, in which the intensity increases with an increase in drug loading. This is further supported by some ^15^N ^1^H dipolar dephasing experiments carried out on the 40 wt % IMC-EE ASD that clearly show this latter signal at −344 ppm with a reduced signal intensity, indicating strong coupling to a proton and hence suggesting a protonated EE amino signal involved in the acid–base interaction with the IMC.

**Figure 5:**
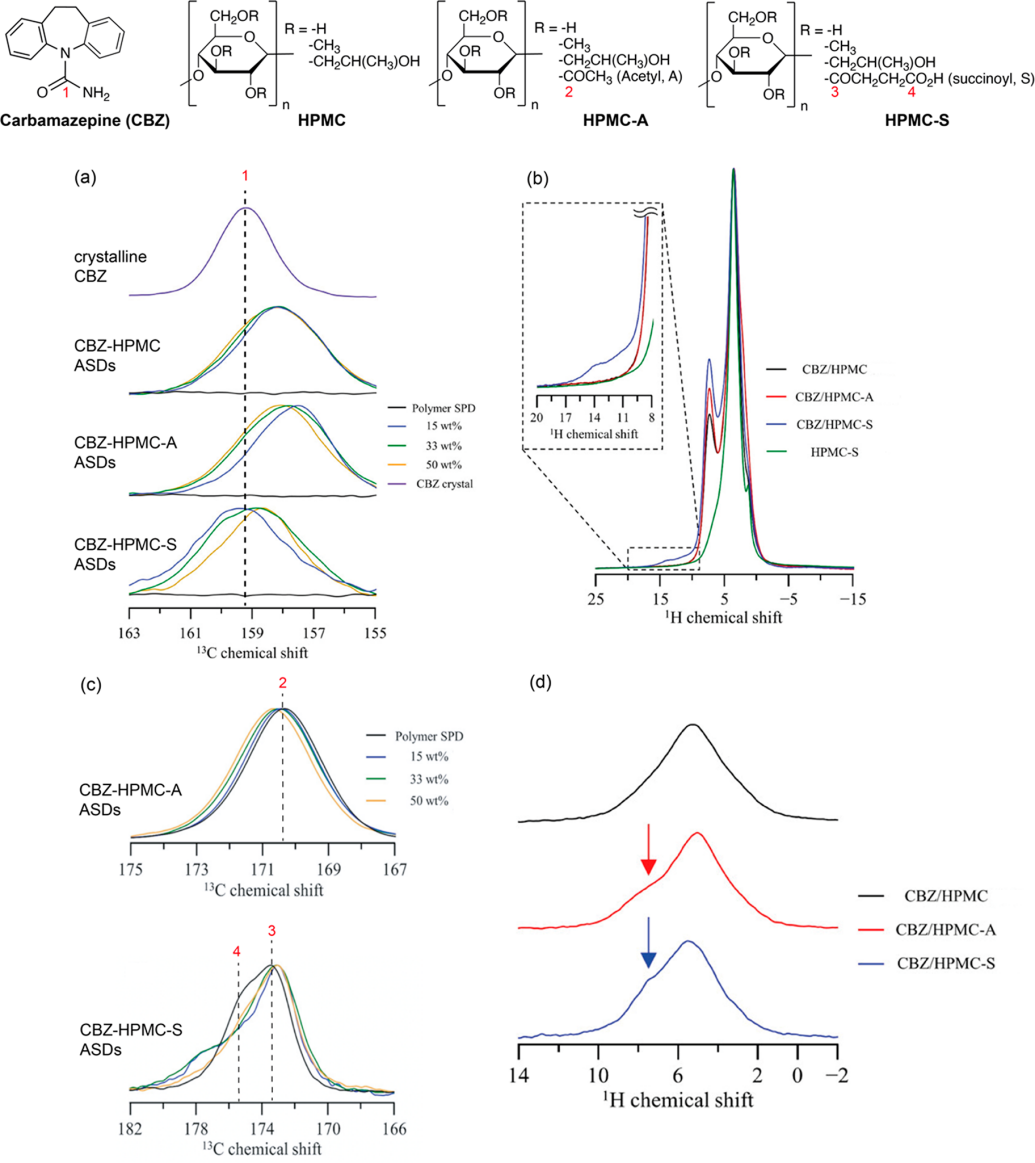
Magnified view of ^13^C CP MAS NMR spectra of the (a) amide region of CBZ, (b) ^1^H ultrafast MAS spectra of 33 wt % CBZ-HPMC, HPMC-A, HPMC-S ASDs, and HPMC-S polymer, and (c) the carbonyl region of the polymers. (d) ^15^N CP-based HSQC filter ^1^H MAS spectra of 33 wt % CBZ-HPMC, HPMC-A, and HPMC-S ASDs. The structures of CBZ, HPMC, HPMC-A, and HPMC-S are given on the top of the figure. Reprinted from (33). Copyright 2019 American Chemical Society.

HPMC-AS has recently been widely used to stabilize APIs in ASDs.^34,35,38,55,56^ This is due to its unique physical–chemical properties such as a high glass transition temperature (around 120 °C) and the presence of both acetyl (A) and succinoyl (S) moieties (Figure 5), which allow for the effective inhibition of drug crystallization from the amorphous dispersion, forming hydrophobic interactions with the drug.^57,58^ There is also evidence that HPMC-AS can stabilize the drug in solution in *in vitro* systems and, potentially, the gastric milieu, further enhancing the bioavailability of the drug by maintaining it at (or even above, at least temporarily) the saturation solubility of the drug.^59−61^

HPMC-AS was initially developed as a polymer for the enteric coating of tablets to prevent the dissolution of tablets and, therefore, the drug in the stomach or upper regions of the small intestine. Different ratios of succinic acid (which confer pH dependent solubility) and acetic acid groups (which hinder solubility) render the different grades of the polymer soluble at different pH ranges with three grades available. The different ratios of these groups may be important in the formation of bonds and interactions with APIs.

The formation of specific API–polymer interactions has been explored in a study in which carbamazepine (CBZ) was formulated with HPMC, HPMC-A, and HPMC-S.^33^ In this work, CBZ-HPMC, CBZ-HPMC-A, and CBZ-HPMC-S were prepared by SD methodology, and the API–polymer contacts were determined from multinuclear 1D NMR spectra (Figure 5).

In crystalline CBZ, the amide C1 signal resonates at 159 ppm in the spectrum and is shifted upon CBZ dispersion in HPMC, HPMC-A, and HPMC-S polymers (Figure 5a). These changes could confirm the amorphous behavior of the dispersions as such changes in chemical shift are consistent with the retention of intermolecular interactions between crystalline and amorphous specie^49^ and can be used as indicatives of API–polymer interactions, which need to be confirmed by further investigations.

More interestingly, evidence of the presence of CBZ–polymer interactions in ASDs can be found by considering both the ^1^H and ^13^C NMR spectra shown in Figure 5b,c, respectively. In contrast with the spectra of HPMC and HPMC-A polymers, the evidence of a signal at 14 ppm in the ^1^H ultrafast MAS (70 kHz) spectrum of 33 wt % CBZ-HPMC-S ASDs together with the shift of the carboxyl succinoyl group C4 signal support the presence of a H-bond between CBZ and HPMC-S.^25^ In a similar way, the carbonyl of the acetyl substituent C2 signal slightly shifts to lower frequencies as the drug load increases, indicating the presence of a H-bond between CBZ and HPMC-A.

Figure 5d shows the ^1^H–^15^N CP-based HSQC filter experiment carried out on 33 wt %^15^N-labeled CBZ-HPMC/HPMC-A/HPMC-S ASDs. All ^1^H spectra are dominated by the polymer signal at around 5.5 ppm; however, an extra signal in the form of a shoulder at around 7.5 ppm is also observed for both CBZ-HPMC-A and CBZ-HPMC-S. The presence of this signal at higher chemical shift corroborates the H-bond between the acetyl carbonyl and the CBZ’s NH_2_ and between both the succinoyl’s carbonyls and the CBZ’s NH_2_ in CBZ-HPMC-A and CBZ-HPMC-S ADSs, respectively. Taken together, these results point out the critical role that the subsistent groups A and S have in forming specific interactions with CBZ.

As previously reported, HPMC itself does not exhibit an excellent ability to promote the formation of a H-bond with CBZ, while the use of α-glycosyl rutin (Rutin-G), a nonpolymeric additive, allows the formation of a CBZ-Rutin-G H-bond and hence leads to a significantly stabilized amorphous CBZ. This outcome is supported by both 1D NMR experiments and 2D heteronuclear correlation NMR experiments as well as quantum mechanical calculations.^62^

Finally, the extraordinary versatility of NMR as an invaluable tool for the determination of the strength and extent of the H-bond has been demonstrated in an exciting work in which felodipine (FEL) API-different polyvinylpyrrolidone (PVP)-substituted polymer ASDs were thoroughly studied.^63^ Supported by changes in ^13^C chemical shifts and spectral intensities, the degree of the FEL-polymer H-bond was ranked in the following order: PVP > poly(vinylpyrrolidone-*co*-vinylacetate) (PVP-VA) > poly(vinylacetate) (PVAc). This result clearly indicates how the nature, strength, and type of different H-bond acceptors influence the formation of effective and strong API–polymer interactions.

## 3 2D Correlation NMR Experiments as a Toolkit to Probe API–Polymer Contacts at the Molecular Level

At the molecular level, the chemical species directly involved in the API–polymer interactions as well as the observation of structural effects can be obtained from 2D NMR approaches. Together with 1D experiments, 2D correlation experiments are well established NMR methodologies and widely used to answer critical pharmaceutical questions. They represent milestones for the development and design of advanced NMR experiments capable of increasing the range of possible information obtained. Indeed, 2D methodologies often allow the direct detection of API–polymer contacts by correlating different nuclei, restoring spectral resolution, and/or reintroducing through-space dipolar coupling interactions averaged by MAS.

In the presence of API–polymer interactions, the electronic distribution surrounding the nuclei is disturbed and changes in chemical shifts can be observed in the 1D spectra. As the atoms involved in these interactions are close in space and interact via dipolar coupling or via spin-diffusion, these nuclei can generate correlation signals in 2D experiments and hence are directly identified. Therefore, the evidence of the change in chemical shifts and of the presence of correlation signals in 1D and 2D experiments, respectively, together with the novel NMR methods capable of estimating interatomic distances, represent the general strategies used to understand the API–polymer interactions in ASDs. This information can be obtained in a reasonable amount of time (approximately a day in total for each ASD), which is an important consideration.

The HETCOR experiment has proven itself to be a useful technique in that respect and enables correlation spectra between heteronuclei commonly present in API (e.g., usually ^13^C but also recently ^19^F) with the ^1^H nucleus. The use of the ^1^H nucleus in one of the spectral dimension enhances the sensitivity of the experiment, while the use of a heteronucleus in the other spectral dimension enhances the specificity.

An empirical estimation of the range of spin diffusion effects occurring in an HETCOR experiment can be obtained according to eq 1 in which *L*, *D*, and *t* are the maximum diffusion path length, the spin diffusion coefficient, and the diffusion time, respectively, while the brackets denote an ensemble average:
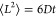
1

The value of the spin diffusion coefficient *D* can be either calculated from eq 2, in which *I*_0_ is the distance between protons in the sample (typically in the range of 0.1 nm) and *T*_2_ is the transverse relaxation time,^41^ or found in the literature from a variety of sources on rigid and mobile polymers (*D* values ranging from 0.5 × 10^–12^ ^64^ to 8.0 × 10^–12^ cm^2^ s^–1^^65^ are available).
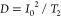
2

For a 500 μs contact time, eq 1 yields an average *L* of about 5 Å as a maximum range over which ^1^H–^1^H spin diffusion can occur within the ^1^H spin-lock period of the contact time in the CP-HETCOR experiment. Regarding the direct dipolar coupling ^1^H–X interaction, its magnitude (transferred by the CP-HETCOR experiment) is limited to the 3–5 Å range.^12^ Experimentally, it has been demonstrated that ^19^F–^13^C HETCOR experiments carried out at contact times of 6 and 8 ms can directly probe API–polymer contacts for spatially close species of 5.5 and 8 Å, respectively.^66^

In addition to the HETCOR experiment, methods based on the detection of homonuclear dipolar coupling, such as ^1^H–^1^H double quantum (DQ) correlation (for example, using the back to back (BABA) recoupling scheme^67^), have been exploited to detect API–polymer proximities in ASDs.

More recently, molecular associations have also been identified via ^14^N–^1^H 2D experiments. Despite the high natural abundance of ^14^N (99.6%), its low gyromagnetic ratio and large quadrupolar interaction make its direct detection in the solid state a challenge, and the development of indirectly detected ^14^N via ^1^H, for example, via 2D ^14^N–^1^H HMQC (heteronuclear multiple quantum coherence)^68,69^ at a high magnetic field (>16.4 T) and ultrafast MAS frequency (>50 kHz), has enabled one to solve this challenge and opened up new possibilities, in particular in pharmaceutical sciences.^38,70−73^

Recent technological advancements have allowed for the development of both magnets that can handle very high magnetic fields and probes that can operate under ultrafast MAS conditions. In particular, this latter progress has benefitted from the significant increase in resolution of ^1^H solid-state NMR spectra by averaging the ^1^H–^1^H mononuclear dipolar interactions and opening a way to access further details on the structure and dynamics information in pharmaceutical formulations.^74^

**Figure 6:**
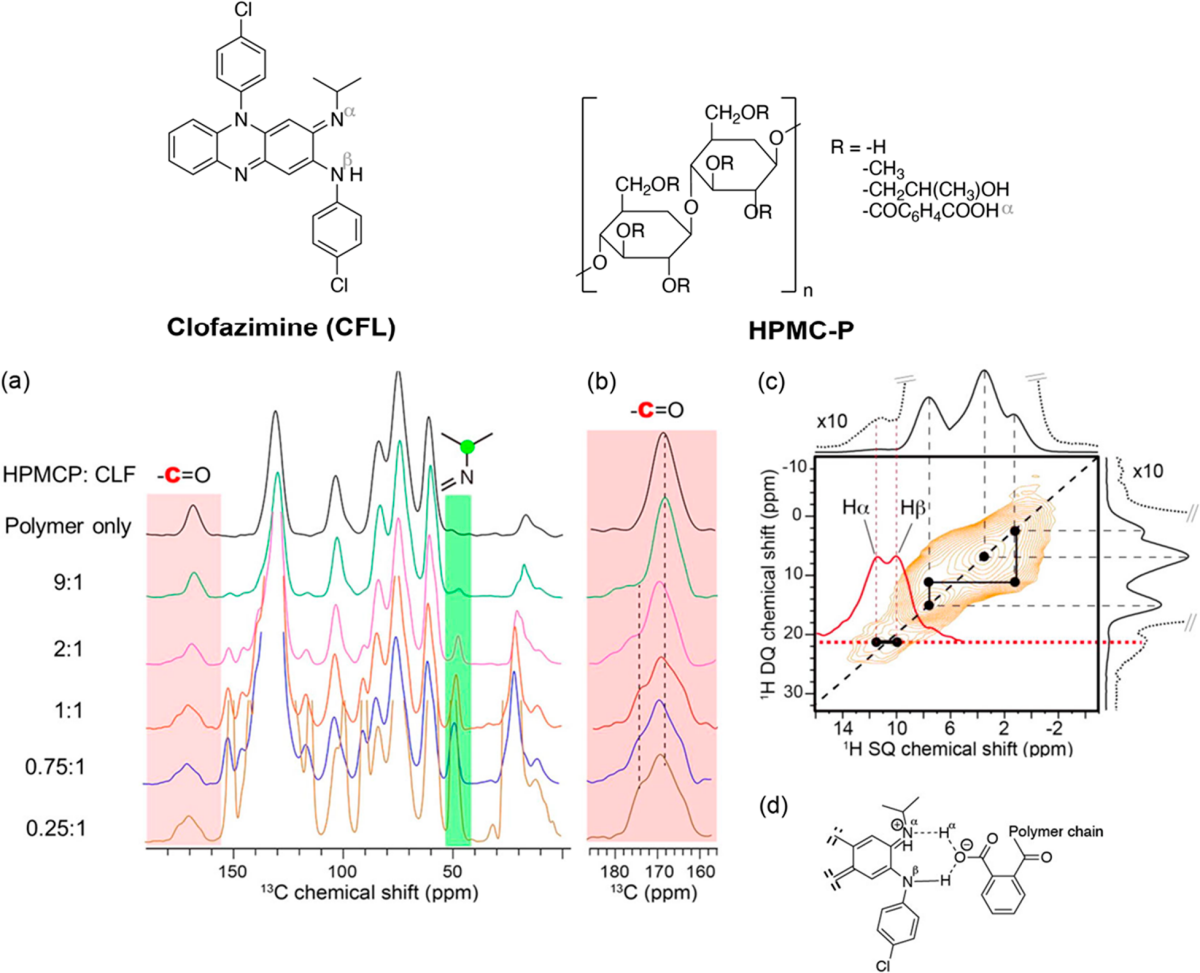
(a) ^13^C CP MAS NMR spectra of CLF–HPMC-P dispersions as a function of API content. (b) Magnified view of the 157–183 ppm region. (c) The ^1^H–^1^H DQ BABA correlation spectrum of 50 wt % CLF-HPMC-PASD exhibits a correlation between Hα and Hβ, indicating the presence of a carboxylate–CNH molecular interaction. The ^1^H spectra of the CLF-HPMC-P dispersion is given on the top. (d) Schematic representation of the API–polymer interaction via proton transfer. The chemical structures of CFL and HPMC-P are given on the top. Reprinted from ref (31). Copyright 2016 American Chemical Society.

Clofazimine (CLF) API-hypromellose phthalate (HPMCS) polymer molecular interactions have been elucidated from 1D and 2D experiments.^31^Figure 6a shows the ^13^C CP MAS NMR spectra of CLF-HPMC-P dispersions as a function of different API loadings. The carboxyl acid in HPMC-P (red in Figure 6a,b) and CHN in CLF (green) signals were used as spy signals to detect the presence of API–polymer interactions, and interestingly, variations in chemical shifts for both signals are observed. The changes in ^13^C chemical shifts observed for the carboxyl acid (from around 170 ppm of HPMC-P to 174.2 ppm in the dispersions) coupled to the one of the CNH in the ASD (by 2.5 ppm against the amorphous CLF) are attributed to the carboxylate group bonding to CLF.

In order to further refine the nature of the molecular interaction occurring in this dispersion, useful and detailed information was obtained from a 2D ^1^H–^1^H DQ BABA experiment on the 50 wt % CFL-HPMC-P ASD. The 1D ^1^H NMR spectrum obtained under an ultrafast MAS condition of 60 kHz (top spectrum in Figure 6c) shows two signals at 11.5 and 10 ppm, which are correlated as revealed from the 2D ^1^H–^1^H DQ BABA spectrum (Figure 6c). Interestingly, these signals have equal peak intensities (see the red solid line in Figure 6c), and their high chemical shifts indicate strong H-bonding of a proton adjacent to a nitrogen atom. Considering the chemical structures of both API and polymer, these two new resonances can be assigned to the existing Nβ–Nα and the newly formed Nα–Hα contacts (see Figure 6d). These molecular interactions indicate a transfer proton mechanism from Hα to Nα with a concerted formation of the interaction between COO^–^ moieties with Nβ.

**Figure 7:**
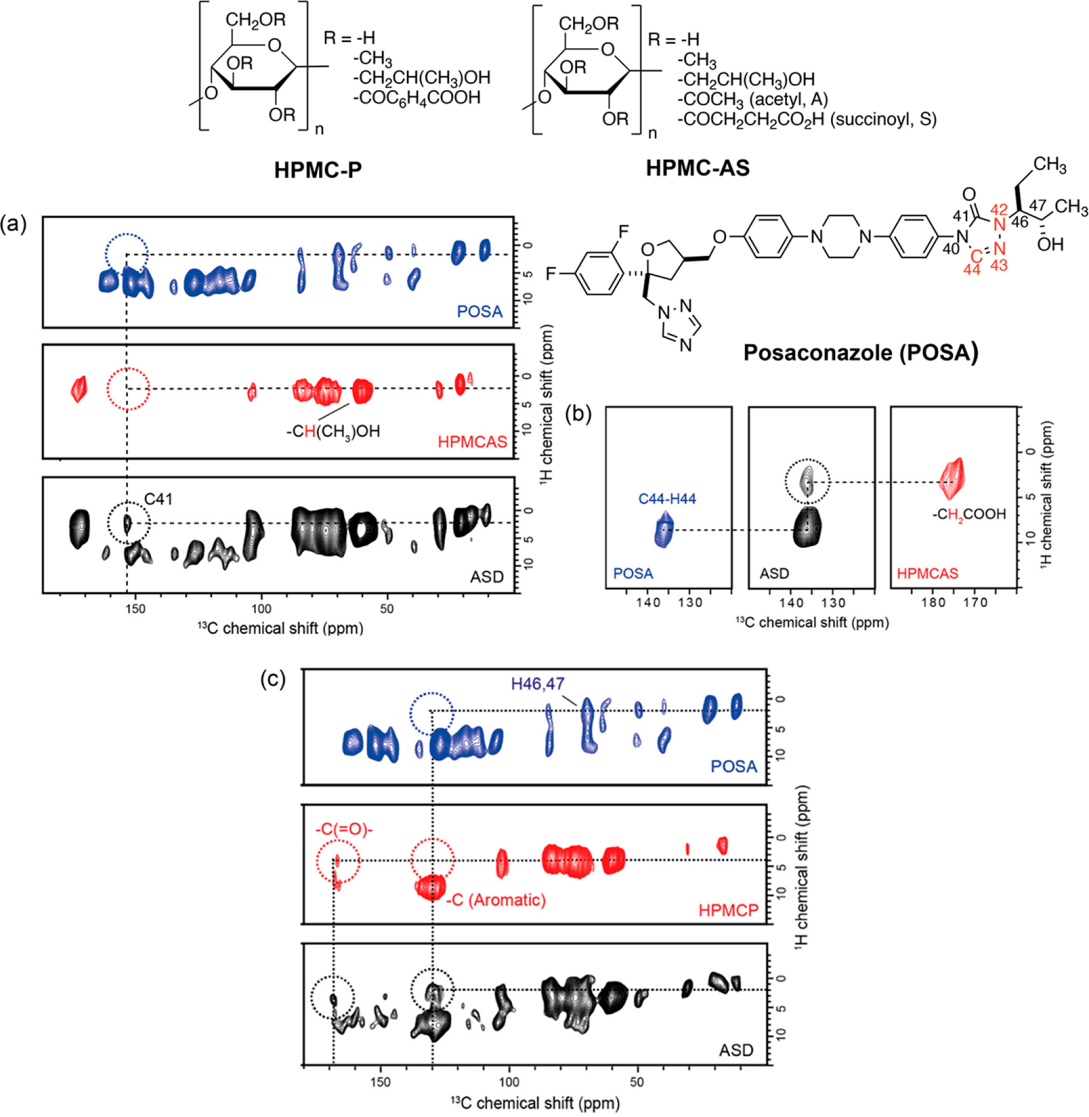
(a) ^13^C CP HETCOR spectra of POSA, HPMC-AS, and 30 wt % POSA–HPMC-AS ASD. (b) ^13^C CP HETCOR spectra of isotopically labeled ^13^C–C44, ^15^N–N42, and ^15^N–N43 sites with POSA, an ASD in HPMC-AS, and HPMC-AS. (c) ^13^C CP HETCOR spectra of POSA, HPMC-P, and 30 wt % POSA–HPMC-AS ASD. The correlations highlighted in the black circle in all ASDs HETCOR spectra indicate the carbon signals involved in API–polymer interactions. All the spectra were carried out at a MAS frequency of 12 kHz with a contact time of 2 ms. Chemical structures of POSA, HPMC-P, and HPMC-AS are given on the bottom of the figure. Isotopically labeled C44, N42, and N43 atoms are highlighted in red. Reprinted from ref (34). Copyright 2019 American Chemical Society.

The capability of HPMC derivatives such as HPMC-P and HPMC-AS polymers to stabilize amorphous APIs in ASDs by forming API–polymer interactions has been also demonstrated with posaconazole (POSA; Figure 7).^34^ The ^1^H–^13^C HETCOR spectrum of 30 wt % POSA–HPMC-AS ASD (Figure 7a) and the correlated ^13^C and ^1^H signals at around 154 and 2 ppm, respectively, demonstrate an intermolecular H-bond interaction between the carbonyl C41 carbon of the POSA triazole ring and the HPMC-AS hydroxyl group. Another electrostatic intermolecular interaction is revealed in the HETCOR spectrum of the 30 wt % isotopically labeled ^13^C–C44, ^15^N–N42, and ^15^N–N43 sites with POSA in HPMC-AS ASD (Figure 7b) from the correlation involving ^13^C at 136 ppm with ^1^H at 4 ppm, which indicates spatial contact of the POSA triazole ring with the −CH_2_ adjacent to the carboxylic moiety in the S group. In a similar fashion, the HETCOR spectrum of 30 wt % POSA–HPMC-P ASD (Figure 7c) highlights two spatial proximities: an electrostatic interaction between the HPMC-S carboxylic group with the POSA triazole ring at 168 ppm/4 ppm and a π–π aromatic stacking interaction between the HPMC-P aromatic carbons and the POSA proton attached to C46/47. These data clearly indicate the role played by the substituent groups of the HPMC backbone, as the presence of hydroxyl and carbonyl groups, triazole moieties, and difluorophenyl rings favor the potential formation of strong interactions with the A and S substituents of HPMC-AS.

**Figure 8:**
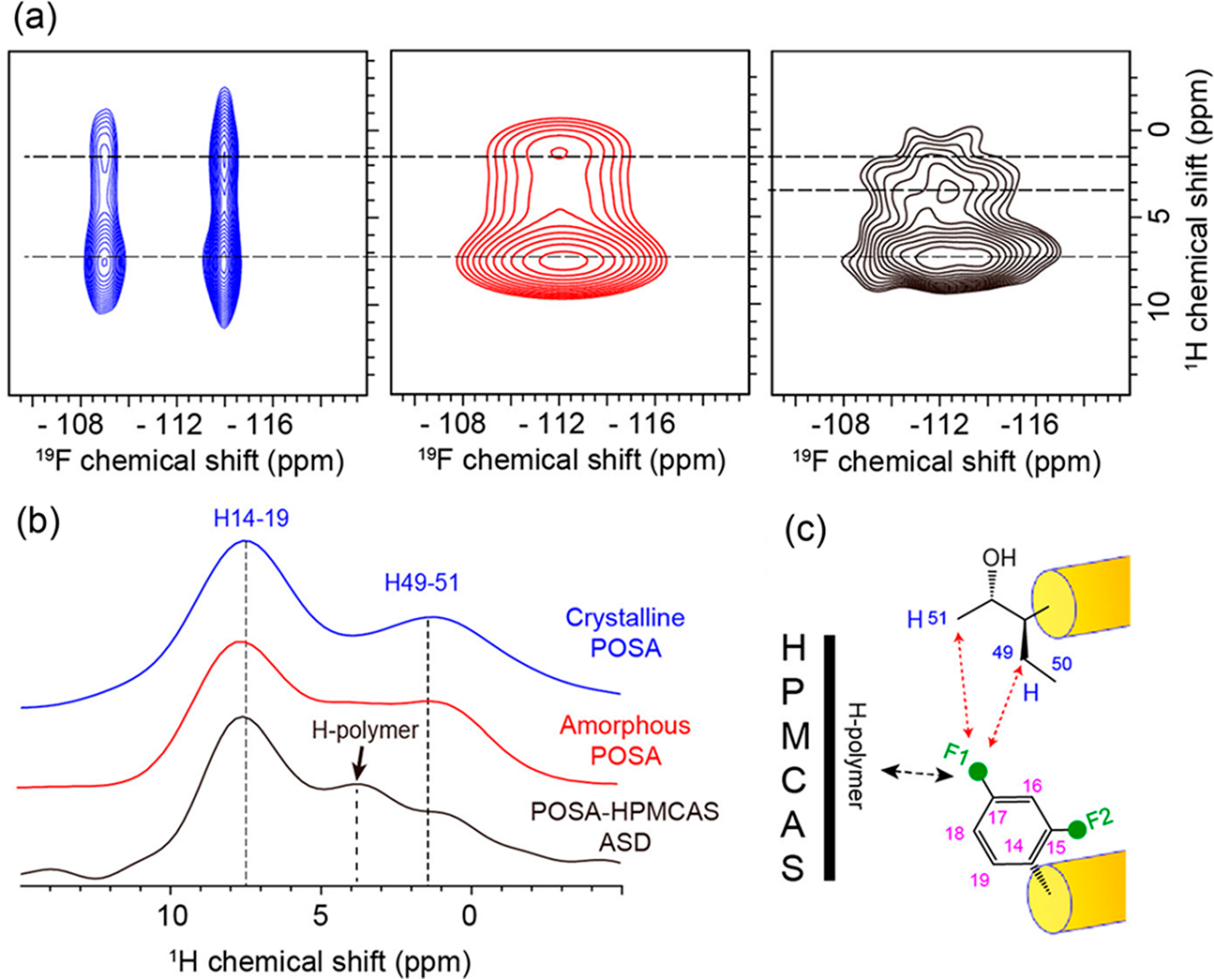
(a) ^1^H–^19^F HETCOR spectra of crystalline (blue) and amorphous POSA (red) and 30 wt % POSA–HPMC-AS ASD (black). (b) ^1^H spectrum extracted from the HETCOR spectra at ^19^F chemical shifts of around −114 (for crystalline POSA), −112 (for amorphous POSA), and −112 ppm (for the ASD), respectively. (c) Schematic representation of intermolecular interactions in amorphous POSA (red dashed arrows) and between POSA and HPMC-AS (black dashed arrow). Reprinted from ref (35). Copyright 2020 American Chemical Society.

The presence of F atoms in the chemical structure of POSA also allows for a further investigation of the presence of API–polymer interactions involving these fluorinated moieties, and exciting outcomes have been reported from the characterization of a 30 wt % POSA-HPMC-AS ASD using 2D ^1^H–^19^F HETCOR.^35^ The corresponding spectra for crystalline and amorphous POSA (blue and red, respectively, in Figure 8a) show similar correlations between the aromatic fluorines of the difluorophenyl group with both aromatic protons (7.5 ppm) and aliphatic protons (1.5 ppm) due to intermolecular “head-to-tail” packing. The extra correlation at around 4 ppm that appears in the HETCOR spectrum of the ASD (Figure 8a,b) additionally suggests the presence of an H-bond POSA-HPMC-AS interaction, likely between the hydroxyl group of HPMC-AS and the difluorophenyl group of POSA, as schematically illustrated in Figure 8c.

A further understanding of the interaction in POSA-HPMC-AS ASD^35^ has also been obtained by measuring the fluorine–carbon interatomic distance using the well-known rotational-echo double-resonance (REDOR) experiment^75^ applied to the ^19^F–^13^C pairs. The REDOR experiment uses rotor-synchronized radiofrequency pulses to reintroduce the MAS averaged dipolar coupling, hence allowing the experimental evaluation of a precise distance between heteronuclear spins. For the POSA-HPMC-AS dispersions, a comparison between experimental dephasing ^19^F–^13^C REDOR curves and simulation yields a close proximity of around 4.3 Å between the HPMC-AS hydroxyl group and the POSA’s difluorophenyl group, adding further important details for the understanding of the POSA-HPMC-AS interaction described above.^35^

^1^H–^13^C and ^1^H–^29^Si HETCORs have been employed to identify spatial proximities and intermolecular interactions between the various components of the IMC-HPMC-mesoporous silica ternary ASD prepared using different HME screw conditions (low- and high-energy HME).^76^ While the ^1^H–^13^C HETCOR data show proximity between IMC and HPMC, the ^1^H–^29^Si HETCOR spectrum provides information on the interaction between IMC-HPMC and the silicon framework. Indeed, IMC/HPMC–mesoporous silica interactions were found in the formulation prepared by the high-energy process.

A further example of the usefulness of the HETCOR technique in the identification of the spatial correlations between API and polymer was given in the stability of the structure of the abiraterone-hydroxypropyl-β-cyclodextrin dispersion^77^ prepared by an innovative solvent-free technology known as KinetiSol.^78^ The ^1^H–^13^C HETCOR showed a clear interaction between the aromatic region of the abiraterone and the anomeric protons of the cyclodextrin.

**Figure 9:**
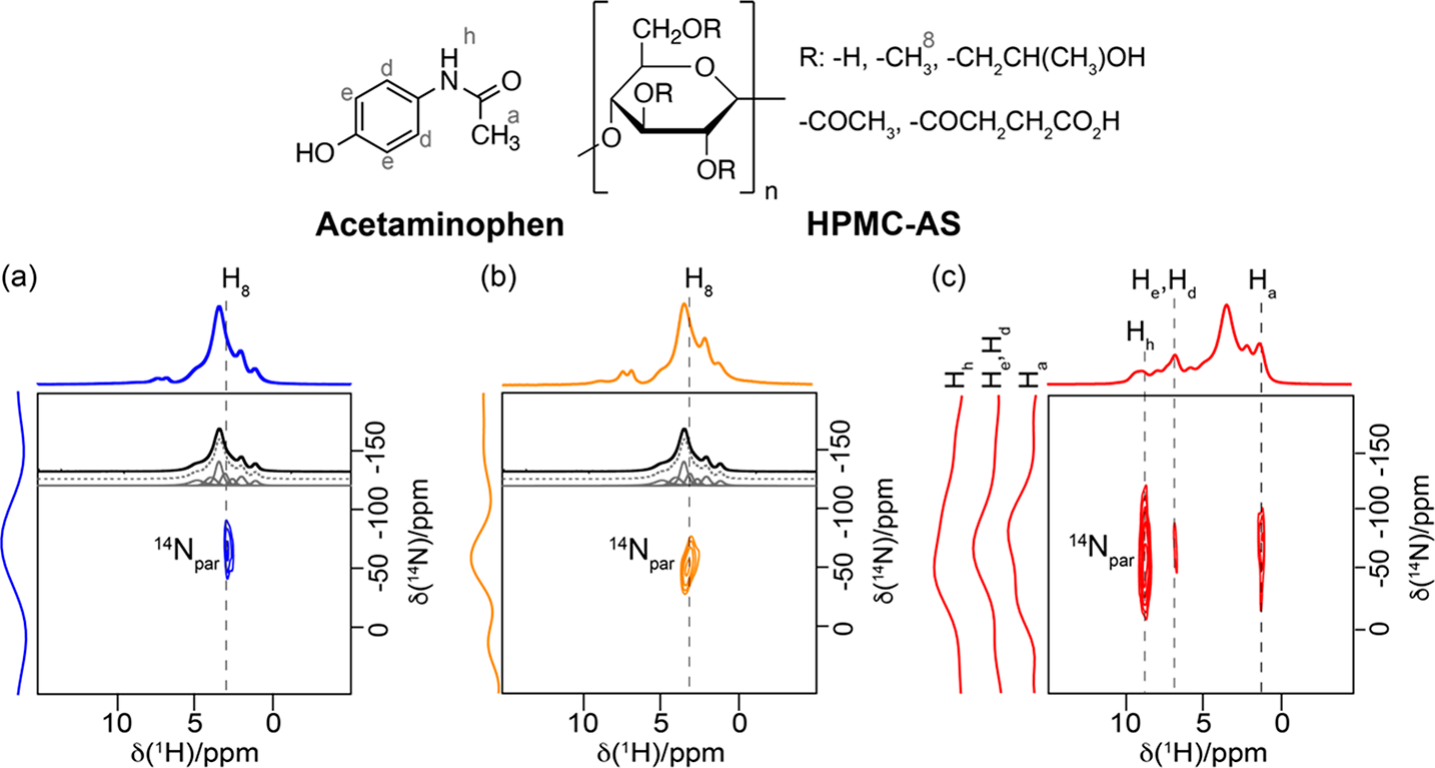
^14^N–^1^H HMQC spectra of 10 wt % (blue), 20 wt % (orange), and 40 wt % (red) acetaminophen-HPMC-AS ASDs. The deconvoluted ^1^H spectra of the HPMC-AS are given in black. Spectra on the left of the 2D HMQC are the ^14^N slices extracted at the indicated ^1^H chemical shift in dashed black lines. Chemical structures of acetaminophen and HPMC-AS are given on top. Reprinted from ref (38). Copyright 2021 American Chemical Society.

The versatility of HPMC-AS in the formation of drug–polymer interactions and, hence, stabilization of the ASDs has also been probed in the acetaminophen-HPMC-AS dispersions (Figure 9).^38^ For the ASDs with drug loading of >20 wt %, ^1^H–^13^C HETCOR correlation experiments identify spatial proximities between aromatic protons of the acetaminophen with the cellulose backbone protons of the HPMC-AS polymer, while the presence of H-bonds between API and polymer was established by the ^14^N–^1^H HMQC experiments. The ^14^N–^1^H HMQC spectra of 10 and 20 wt % acetaminophen-HPMC-AS ASDs (Figure 9) indicate the presence of interactions between the acetaminophen ^14^N signal with the −OCH_3_ signal (H_8_) of the polymer, hence highlighting a closer contact between API and the polymer, which can be reasonable attributed to the presence of a H-bond between this amide donor and oxygen acceptor. In contrast, the ^14^N–^1^H HMQC spectrum of the 40 wt % ASD exhibits correlations between the acetaminophen ^14^N signal with all acetaminophen protons in the crystalline form, suggesting the absence of the API–polymer interaction, instability, and API recrystallization with loading of >40 wt %.

**Figure 10:**
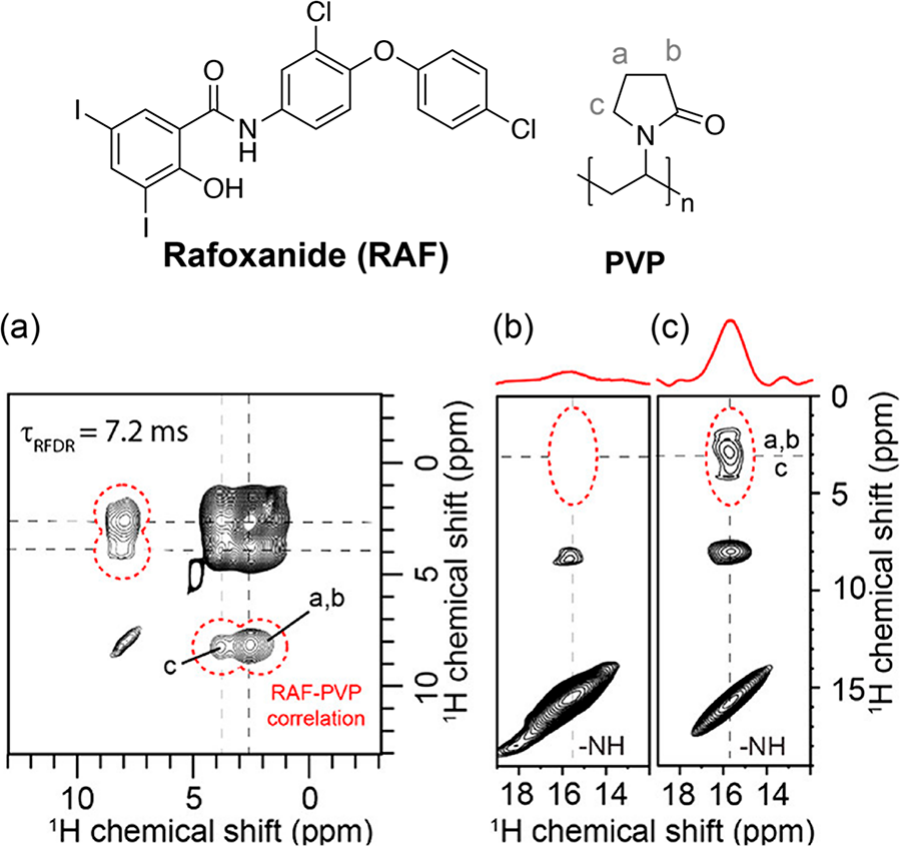
(a) ^1^H–^1^H RFDR spectra of 50 wt % RAF-PVP ASD prepared via SD under aqueous conditions carried out at mixing time (τ_RFDR_) of 7.2 ms. Enlarged region of ^1^H–^1^H RFDR spectra carried out at (b) low and (c) very-high magnetic field, showing the evidence of the presence of the RAF-PVP H-bond. Correlation peaks are highlighted with a red dotted line. Chemical structures of RAF and PVP are given on the top. Reprinted from ref (37). Copyright 2020 American Chemical Society.

The evaluation and the evidence of the API–polymer interactions in Rafoxanide (RAF)-PVP ASDs at drug loadings of 25, 33, and 50 wt % (Figure 10), prepared via SD using two different feed solutions, aqueous (a 70%/30% mixture of 0.1 M NaOH solution and acetone) and organic (80%/20% acetone/ethanol mixture), have been probed using 1D ^13^C and ^15^N CP experiments and further investigated carrying out 2D ^1^H–^1^H RFDR (radio frequency driven recoupling)^37^ experiments at a MAS of 110 kHz at 18.8 T (Figure 10), which leads to the determination of ^1^H–^1^H proximities by recoupling homonuclear dipolar interactions. For the 50 wt % RAF-PVP ASD made from aqueous conditions, the spectrum recorded at a short mixing time (τ_RFDR_) of 7.2 ms (Figure 10a) shows the correlation between the PVP aliphatic protons (2–3.5 ppm) with the RAF aromatic protons (ca. 7.5 ppm) and, more importantly, exhibits long-range intermolecular correlations between RAF aromatic protons and PVP aliphatic protons (peaks circled in red). Moreover, thanks to the enhanced ^1^H resolution likely due to the benefit of a very-high magnetic field together with the ultrafast MAS condition, the enlarged RFDR spectrum (Figure 10c) illustrates the presence of cross peaks between the amide ^1^H of RAF with the aliphatic ^1^H of PVP and highlights the expected amide–aromatic proton intramolecular correlation, indicating the presence of an API–polymer intermolecular H-bond interaction involving RAF and PVP. Reasonably, the formation of this interaction is due to the aqueous feed solution used during the SD process. In the aqueous condition, the presence of the NaOH can ionize the RAF phenolic hydroxyl group to promote the formation of a H-bond between the RAF amide, as a donor, and the PVP carbonyl, as an acceptor. The presence of this RAF-PVP H-bond interaction for these dispersions has also been confirmed from ^1^H–^13^C HETCOR.^79^

## 4 Conclusions

In this Review, we have described the latest applications of solid-state NMR spectroscopy approaches employing 1D and 2D data acquisition strategies to identify and provide an understanding of the API–polymer interactions in pharmaceutical formulations. These studies revealed that changes of ^1^H, ^13^C, and ^15^N chemical shifts in ASDs when compared with their individual components allowed the identification of API–polymer interactions such as a H-bond or ionic one, while correlation spectroscopy from 2D NMR spectra has proved itself to be an excellent tool for molecular level identification of chemical species involved in the API–polymer contacts, providing essential information to understand the nature of the stabilizing interactions.

Moreover, to strengthen the understanding of the stability mechanism of the ASDs, NMR methodologies can play an important role as an orthogonal approach to the various more commonly used methods such as UV, IR, and XRD.^28,30,31,33^ Furthermore, NMR spectroscopy can be an effective and a powerful technique to aid the pharmaceutical scientist in the design of new potential ASDs.

The continuous development of NMR leads to the design of increasingly sophisticated and sensitive methods, and this will certainly open up opportunities to understand ASD systems and, therefore, allow the rational design of appropriate systems, rather than the current empirical approach. The dynamic nuclear polarization (DNP) technique, thanks to its enhanced sensitivity compared to traditional NMR, has been successfully applied to the field of pharmaceutical sciences. Pioneering works that explore the dependence of DNP enhancement on sample composition, radical concentration, relaxation properties of the API, excipients, and formulations, types of polarizing agents, and proton density have been published and offer exciting perspectives.^80,81^
